# Growth Rate Distribution and Potential Non-Linear Relationship between Body Weight and Walking Ability in Turkeys

**DOI:** 10.3390/ani13182979

**Published:** 2023-09-20

**Authors:** Samet Soyalp, Evan Hartono, Owen W. Willems, Xuechun Bai, Benjamin J. Wood, Samuel E. Aggrey, Romdhane Rekaya

**Affiliations:** 1 Department of Animal and Dairy Science, University of Georgia, Athens, GA 30602, USA; 2 Department of Poultry Science, University of Georgia, Athens, GA 30602, USA; 3 Hybrid Turkeys, Kitchener, ON N2K 352, Canadaxuechun.bai@hendrix-genetics.com (X.B.); 4 School of Veterinary Science, The University of Queensland, Brisbane, QLD 4343, Australia; b.j.wood@uq.edu.au

**Keywords:** turkeys, walking ability, growth rate, distribution

## Abstract

**Simple Summary:**

Intense selection has led to a significant increase in body weight of commercial turkeys. These successes have had some impact on the mobility of turkeys. There is a consensus about the negative genetic relationship between growth and mobility. Although it is reasonable to assume that such a relationship is likely to change with the rate of growth, little is known about the potential non-linearity of such association. To investigate the potential non-linear relationship between growth and mobility, the growth rates at three different age periods (0 to 12, 12 to 20, and 0 to 20 weeks) were calculated, and each bird was assigned to one of the quartiles of the growth rate distribution for each age period. Heritability estimates of walking ability ranged between 0.18 and 0.26 indicating the possibility for genetic improvement. The genetic correlations between adjacent growth rate quartiles were high and decayed as the interval between quartiles increased. There is no evidence to support that walking abilities across growth rate quartiles are different traits. However, the magnitude of the variation in the incidence of walking scores and genetic correlations across the different growth quartiles seem to point towards a potential non-linear relationship between growth and mobility.

**Abstract:**

The aim of this study was to investigate the potential non-linear relationship between growth and walking ability (WA). The phenotypic data included body weights at 12 and 20 weeks and WA at 20 weeks of age measured on 276,059 male turkeys. The growth rate at three age periods (0 to 12, 12 to 20 and 0 to 20 weeks) was calculated. Each bird was assigned to one of the quartiles of the growth rate distribution for each age period. Between the first and fourth quartiles, the incidence of score 1 (bad WA) increased by 31, 18, and 33% for the first, second, and third age periods, respectively. For good WA (scores 4, 5, and 6), the incidence decreased by 55, 66, and 72% between the first and fourth quartiles for the first, second, and third age periods, respectively. Estimates of heritability of WA ranged between 0.18 and 0.26. The genetic correlations between adjacent growth rate quartiles were high and decayed as the interval between quartiles increased. The magnitude of the variation in the incidence of walking scores and genetic correlations across the growth rate quartiles point towards a non-linear relationship between growth and mobility suggesting other factors may affect walking ability.

## 1. Introduction

Enormous progress has been achieved in production and feed efficiency traits in turkeys due to genetic selection. These successful breeding programs have resulted in a significant decrease in feed conversion rate, reduced age to slaughter, and increased breast meat yield. This success has led to some unintended consequences including decreased reproductive performance, skeletal abnormalities, and increased carcass fatness [1]. The significant reduction in the number of days to market weight and the disproportional increases in breast meat yield has resulted in the increase in the incidences of various skeletal abnormalities, leg weakness, and locomotion problems.

Walking ability is one of the indicator traits used by the poultry industry to assess bird mobility and to correct some of the leg related abnormalities. It is a complex trait caused by multiple factors [2,3,4]. Bacterial chondronecrosis, valgus-varus deformity, tibial dyschondroplasia, and contact dermatitis are the most frequent anomalies leading to walking ability problems [2,5,6]. Birds with compromised mobility tend to have reduced ability to carry out routine functions including the ability to reach feeders and drinkers in extreme cases [2,3,4,7,8]. Consequently, the ability of the bird to move has a direct association with productivity and animal welfare. Walking ability is subjectively scored based on a six-grade scoring approach, with 1 being poor and 6 being good. Motion, pitch, balance, leg angulation, hock strength, hip strength, and leg structure are the seven mobility features used to subjectively assess walking ability scores. Quinton et al. [9] present a full description of the scoring system.

Although there is an extensive body of evidence about the likely negative relationships between growth traits and walking ability [10,11,12,13], there is no consensus based on published data [14]. In fact, reported genetic correlation estimates between body weight and walking ability traits ranged between −0.73 and 0.51 [9,10,14,15]. Some of the large variation, both in direction and magnitude, in the genetic association between growth traits and walking ability reported across studies can be attributed to differences in trait definition, sample size, and methodology used for data processing and analysis. Some biological factors related to the nature of the association may also play a role.

The assumption that such relationships are homogeneous across the growth gradient, as assumed in all the reported studies, may not be true. Furthermore, it may have a direct impact in the genetic evaluation of walking ability when growth rate is present in the analysis as a correlated trait or as a covariate. It is reasonable to postulate that the relationship between growth and walking ability may not be constant as a function of the rate of growth. The assumption of non-linearity of the relationship between growth and walking ability is biologically well-supported as mobility problems are less likely to occur until the bird’s body weight (growth rate) exceeds a certain threshold. The aims of this study are (1) to investigate the potential non-linearity in the relationships between growth rate and walking ability using multi-trait threshold model, and (2) to assess the impact of such non-linearity on the ranking of candidates for selection.

## 2. Materials and Methods

All the data used in this project were previously collected, and an Animal Use and Care Protocol was not needed. Data were provided by Hendrix Genetic (Canada) and consisted of phenotypic and pedigree information on turkeys hatched between 2009 to 2018. The phenotypic data was collected on 276,059 birds. The pedigree consisted of 836,785 birds. Three traits (body weights at 12 and 20 weeks and walking ability at 20 weeks) were used in this study. All birds were scored for walking ability using the 6-class (1 to 6) scoring system. A score of 1 indicates an extreme lateral deviation, and scores of 2 and 3 indicate an intermediate lateral deviation of the legs. Scores of 4, 5, and 6 indicate slight to no lateral deviation of the legs. Due to the small number of animals with scores of 4, 5, and 6, birds with these scores were joined into one class (class 4). After this change, walking ability was classified based on 4 scoring classes. After editing, the data consisted of 75,094 birds recorded for 3 traits: body weight at 12 weeks (BW12), body weight at 20 weeks (BW20), and walking ability at 20 weeks (WA). There were 27,753 (37%), 31,153 (41%), 14,554 (19%), and 1634 (3%) birds with walking ability scores of 1, 2, 3, and 4, respectively.

Growth rates were computed for all birds across three time periods (Period 1 = 0 to 12 weeks; Period 2 = 0 to 20 weeks; and Period 3 = 12 to 20 weeks). Based on the distribution of growth rate within each of the three periods, birds were clustered into one of four classes (1 = 1st quartile; 2 = 2nd quartile; 3 = 3rd quartile; and 4 = 4th quartile). Walking ability within each of the four classes of growth distribution for each period was used to assess its relationship with growth rate. Summary statistics of the data were obtained using different packages of R [16].

For each period, walking ability was considered as different categorical response within each of the four growth rate classes. A multivariate threshold model was used to jointly analyze the 4 discrete traits for each period separately in order to assess their genetic parameters. At the liability scale, the following model was used:

(1) $$l_{ijk}=H_{ik}+u_{jk}+e_{ijk}$$

where $l_{ijk}$ was the non-observed liability for walking ability in growth rate class $k\;\left(k=1-4\right)$ for bird *j*, $\;H_{ik}$ was the fixed effect of the hatch week class *i* for trait *k*,$\;u_{jk}$ is the random additive effect of bird *j* for trait *k*, and $e_{ijk}$ is the random residual term.

Assuming normality conditionally on the model parameters, the joint distribution of the liabilities can be expressed in matrix notation as:

(2) $$l\;|\;\beta ,\;u,\;R0\;\sim \;N\;\left(X\beta +Zu,\;R0\;⊗\;I\right)$$

where $l=\left(l_{1}^{,},\;l_{2}^{\prime },l_{3}^{\prime },\;l_{4}^{,}\right)\prime$ is the vector of liabilities for the four traits, *β* and u are vectors of systematic and random effects, respectively; and R0 is a 4 × 4 residual (co)variance. *X* and *Z* are known incidence matrices with the appropriate dimensions.

Because a given bird will have a walking ability score only in one of the four growth rate classes for each time period, the residual covariances between traits cannot be estimated, and they were set to zero resulting in a diagonal matrix R0.

Flat bounded priors were assumed for the fixed effects, and classical multivariate normal distributions for the additive effects ($p\;(u\;|\;A,\;G)\;\sim \;N\left(0,\;G0⊗\;A\right)).$ For the genetic (G0) and residual (R0) covariance matrices, flat bounded priors were used. The resulting full conditional distributions needed for the implementation of Gibbs sampling for the systematic and random effects, liabilities, and genetic covariance matrix, and the diagonal element of R0 were in closed form being normal, truncated normal, scaled inverse Wishart, and scaled inverse Chi Square distributions, respectively. A unique chain of 100,000 samples was implemented, where the first 30,000 samples were discarded as burn-in period based on visual inspection of the behavior of the chain. Necessary analyses were carried out using Fortran computer programs previously developed and used by our team [17,18].

To further investigate the potential non-linear associations between growth rate and walking ability, growth rates within the three time periods (Period 1 = 0 to 12 weeks; Period 2 = 0 to 20 weeks; and Period 3 = 12 to 20 weeks) were analyzed jointly for each walking ability class. Four multi-trait (3 traits) analyses were carried out via classical mixed linear model using the computer software developed by Rekaya et al. [17].

## 3. Results

Table 1 presents a summary of the growth rate distribution across the three age periods. From the lowest to the highest quartiles, the growth rate increased by approximately 24% from 0.080 to 0.099 kg during the first period (0 to 12 weeks). The coefficient of variation ranged between 1 and 4%, with the lowest values corresponding to the intermediate quartiles indicating a sharp distribution with relatively heavy tails. Similar trends were observed for the growth rate distributions for Periods 2 (12 to 20 weeks) and 3 (0 to 20 weeks). The difference in growth rate between the top and bottom quartiles was 38 and 24% for the second and third periods, respectively. The highest coefficients of variation were observed, as expected, for the top (5%) and bottom (8%) quartiles in the second period (Table 1).

The incidence rates of the walking ability scores across the different quartiles of the growth rate distribution across the three age periods are presented in Table 2. For the first period and within a growth rate quartile, class 2 (score 2) has the highest incidence and it ranged between 41 and 41.8%. The lowest incidence was observed for class 4 (original scores of 4, 5 and 6) with an incidence rate ranging between 1.3 and 2.9%.

For the second and third periods (Table 2), a similar trend was observed compared to Period 1. Except for class 2 (walking score of 2), there were significant differences in the incidence of walking ability scores across the growth rate quartiles and age periods. The incidence of class 1 (score 1) increased by 18% and 33% between the first and fourth quartiles for the second and third periods, respectively. However, the incidence of good mobility scores (class 4) decreases by 66% and 72% between the first and fourth quartiles for the same two periods (Table 2), although it should be noted that the prevalence of WA class 4 across the three time periods was low.

Table 3 presents the posterior means of the residual variances for WA across the different growth rate quartiles for the three growth periods. There was little to no difference in the estimates for the second and third quartiles. However, there was a 7.6% increase and 4.6% decrease in the residual variance between periods 1 and 3 for the first and fourth quartiles, respectively. Based on the high posterior density intervals, only the 7.6% increase was statistically significant.

Estimates of heritability and genetic correlations, using a multivariate threshold model, between walking ability classes across the four growth rate quartiles for the three periods are presented in Table 4. Across the different growth rate quartiles and periods, the heritability of walking ability ranged between 0.18 and 0.26.

The highest heritability was observed for the fourth growth quartiles across periods. There was higher similarity between the estimates of heritability of walking ability between the first and second growth rate quartiles and between the third and fourth quartiles. The genetic correlations between adjacent growth rate quartiles for walking ability were always high (>0.84) and decayed as the interval between quartiles increased (Table 4). The highest genetic correlation, across periods, was between first and second quartiles (0.89 to 0.91) and between the third and fourth quartiles (0.94). The lowest correlations were between walking ability scores in the first and forth quartiles of the growth rate distributions and ranged from 0.84 to 0.88.

**Figure 1:**
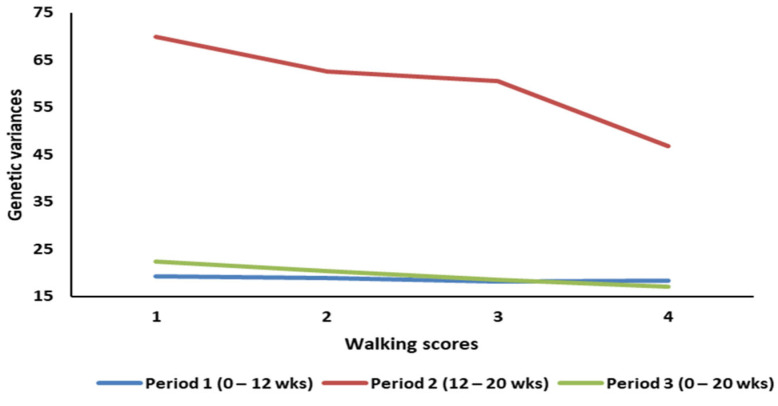
Genetic variance of growth rate across walking ability classes and age periods.

**Figure 2:**
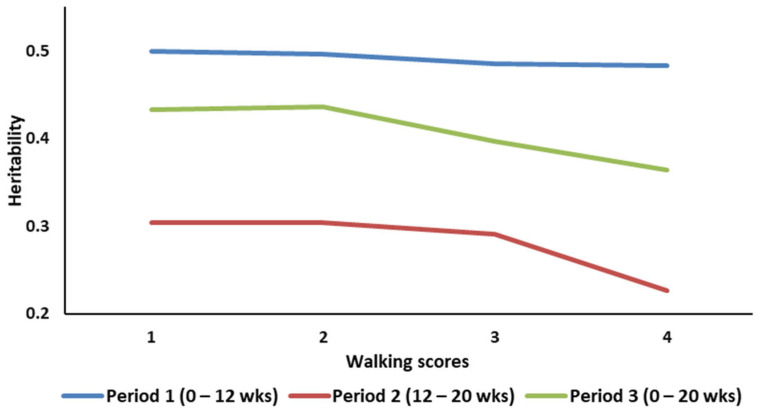
Heritability of growth rate across walking ability classes and age periods.

The average growth rate across the four walking ability classes for the three age periods showed a significant increased with age. Within a growth period, average growth rate decreased with the increase in WA scores. In fact, growth rate decreased by 3, 5, and 6% between WA classes 1 and 4 for Periods 1, 2, and 3, respectively. Within a growth period, the genetic variances for growth rate were similar between adjacent WA classes. However, the genetic variance decreased by 5, 33, and 24% between WA classes 1 and 4 for the periods 1, 2, and 3, respectively (Figure 1). Heritability estimates across the three periods and WA classes are presented in Figure 2. The trend is similar to the one observed for the genetic variances largely due to the small variability in the estimates of the residual variances. The genetic correlation between growth rates across the different WA classes ranged from 0.96 to 0.99, 0.89 to 0.98, and 0.92 to 0.99 for periods 1, 2, and 3, respectively. The genetic correlations between growth rates in adjacent WA classes were high (>0.95) and decayed as the interval between scores increased. The highest genetic correlation, across periods, was between growth rates in WA classes 3 and 4. The lowest correlations were between growth rates in WA scores 1 and 4.

**Figure 3:**
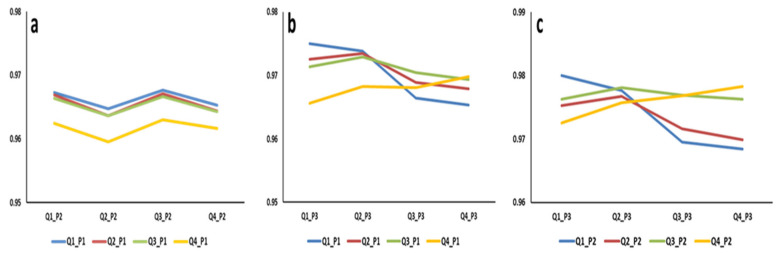
Correlation between breeding values for walking ability across growth quartiles for (**a**) Period 1 (P1; 0–12 weeks) and Period 2 (P2; 12–20 weeks), (**b**) Period 1 and Period 3 (P3; 0–20 weeks), and (**c**) Period 2 and Period 3. Qi_Pj indicates growth quartile i and Period j.

The Spearman correlations between the estimated breeding values of walking ability at the liability scale across the different periods and growth quartiles are presented in Figure 3. Across the different scenarios, the correlation was high and ranged between 0.96 and 0.98. It is higher between the same quartile in different periods and between adjacent quartiles within a period. Across periods, the lowest correlations were observed between the first (0–12 weeks) and second (12–20 weeks) period (Figure 3a). The highest correlations were observed between the second (12–20 weeks) and third (0–20 week) periods (Figure 3c). The lowest correlation (0.96) was observed between the breeding values in the fourth quartile of the first period and the breeding values in the second quartile of the second period (Figure 3a), and the highest (0.98) was between the breeding values in the first quartile of the second and third periods (Figure 3c). The Spearman correlations between the breeding values of walking ability across the different scenarios seem to indicate limited re-ranking.

## 4. Discussion

Across the growth quartiles and except for class 2, there were clear differences in the incidence of the different WA classes. In fact, there was a 31% increase in the incidence of class 1 (score 1) and a 55% decrease in the incidence of class 4 (scores 4, 5, and 6) between the first and fourth quartiles (Table 2). Similarly, there was a 36% decrease in the incidence of class 3 (score 3) between the first and the fourth quartiles. Across the three growth periods, over 70% of the birds had scores of 1 and 2, indicating that the majority of the birds have some level of mobility problems (Table 2). There were very few animals with a walking ability score greater than or equal to 4. Across the different growth periods, there are still enough birds (15 to 20%) with high growth rate (third and fourth growth quartiles) and fair to good walking abilities. Thus, there are reasonable opportunities to further select for growth without additional deterioration of walking ability. Several studies showed a decline in walking ability with the increase in turkeys’ age and weight [19,20,21,22]. Kappel et al. [14] reported small unfavorable genetic correlations between body weight and valgus and varus deformities, and gait scores. That was not the case for a lightweight line of turkeys. Rasmussen et al. [21] showed a significant change in gait scores at three ages (21, 27, and 33 days) in broiler chickens. Although consistent in trend with our results, these studies did not assess the impact of growth rate distribution at a fixed age on walking ability, as was the case in this study. Similar patterns were observed for growth rate across the different WA classes. In fact, growth rate decreased by 3 to 5% between WA classes 1 and 4 across the three periods. In spite of the decline in growth rate, ample genetic variation remains in the population to selection for growth rate while at least avoiding further decline in walking ability.

Collectively, these results show that selection for growth rate across the different age periods may have affected mobility in turkeys. This is likely due a larger allocation of nutritional resources to support breast meat yield than bone and skeleton development. As growth rate increases, the incidence of bad walking ability (score 1) increased by around 25%, and good mobility scores (4, 5, and 6) decreased by more than 50%. These results are in concordance with those reported by previous studies [12,13,23,24]. Although the patterns of incidence of the different walking scores are the same across the different growth rate quartiles and periods, the magnitude of the variations are not similar, indicating a likely non-linear relationship between growth and walking ability. This suggests that there may be other factors affecting walking ability in addition to growth rate.

Estimates of heritability between walking ability classes across the four growth rate quartiles for the 0–12, 12–20, and 0–20-week periods using a multivariate threshold model showed non-negligible variation due mainly to changes in genetic variances across growth quartiles. However, heritability estimates (0.18–0.26) across growth quartiles and period are consistent with reported estimates in the literature [9,13,25]. Our estimates were higher than those reported by [14] for gait scores, tibial dyschondroplasia foot dermatitis, and valgus and varus deformities (0.03–0.15) but almost identical to heritability estimates for WA (0.18–25) obtained by Quinton et al. [9]. This moderate heritability clearly suggests that there is some room for genetic improvement in walking ability. Nestor and Anderson [26] reported that unimproved turkeys selected for increased shank width showed good walking ability and improved leg structure. It is worth mentioning that there was higher similarity between the heritability estimates of WA between the first and second growth rate quartiles and between the third and fourth quartiles; this was likely due to a higher consistency between these phenotypes. These results are in complete concordance with those reported by [25] when WA was classified as a binary trait by joining scores 1 and 2 into one class and scores 3 and 4 into another class.

When growth rate was classified as different traits across the four WA classes, there was a clear difference in the magnitude and patterns of the heritability of growth rate across the four WA classes (Figure 2). Heritability estimates across the three periods and WA classes ranged between 0.22 and 0.50. These estimates are similar to those reported by Soyalp et al. [25], Aslam et al. [27], and Abdalla et al. [28]. However, they tend to be higher than the estimates reported by Kappel et al. [14], where heritability of body weigh ranged between 0.21 and 0.25 for three lines of turkeys. For Period 1 (0 to 12 weeks of age), there was a high consistency in the heritability with only a slight decrease with the increase in WA scores. As the growth rate changes (Periods 2 and 3), there were significant differences in the estimates of the heritability of growth rate across WA score classes. This seems to indicate that as the birds become older and their growth rate accelerates, the association between mobility and growth changes in a non-uniform manner across the different WA classes likely pointing towards a non-linear relationship between growth and WA. The differences in the heritability of growth rate across the different walking ability classes (Figure 2) are largely due to a reduction in the genetic variance. The smallest genetic variances and heritabilities for growth were observed for the fourth walking ability class. This may indicate, on top of the reduced genetic variability in growth between these birds, a scale effect due to smaller body weight for birds with a walking score of 4 and higher.

Estimates of the genetic correlations between the WA scores across growth quartiles and periods were always greater than 0.84, indicating they are genetically similar but not identical. The highest correlations were between walking abilities in adjacent growth quartiles and decreased as the interval between quartiles increases. This is somewhat expected due to the higher similarity in growth rate between adjacent quartiles. Overall, there were substantial changes in the magnitude of the genetic correlation (2 to 12%) between WA across growth quartiles and age periods indicating a potential non-linear effect of growth on turkey’s mobility. Although there are no estimates in the poultry literature to compare to, some studies in human reported a similar trend of non-linearity in the association between leg strength and gait speed [29]. The highest changes in the genetic correlations between growth rates within WA classed were in Period 2 (12–20 weeks). This age period has the highest mean and variability in growth rate across the four WA classes, which is likely the reason for the more pronounced change in the genetic correlations. It is reasonable to speculate that the non-homogeneity in the genetic correlation between growth rate and walking ability may be even more pronounced at the commercial level, where the growth rate distribution is wider and more heterogenous due to increased variability in the management and environmental factors compared to breeding farms.

Our results show that walking ability in turkeys decreased with the increase in growth rate at three age periods. These results are in agreement with those reported in the literature regarding the relationship between WA and age [30,31,32]. The decrease in walking ability with age was associated with the increase in body weight and stocking density [33,34,35]. However, in all these studies, the effects of age and body weight were confounded. Furthermore, these studies did not attempt to estimate the genetic correlation between walking ability and growth rate at a specific age or age period. To the best of our knowledge, this the first study to directly assess this correlation and to try to dissect its potential non-homogeneity across growth rate classes. The results support the hypothesis of a potential non-linear relationship between walking ability and growth rate evidenced by a non-uniform change in the genetic correlation for WA across growth quartiles and age periods. A possible explanation for this non-linearity might be the difference in resource allocation for skeleton development and growth between birds with different growth rate. A similar non-linear relationship between leg strength and gait speed was reported in human [29].

The evidence from this study suggests that the effect of growth rate on walking ability may vary in a non-linear fashion. The significant changes in the incidence of the different mobility problems across periods and growth quartiles and the moderately high estimates of the genetic correlations between walking ability scores across the different growth rate quartiles and periods seem to point toward some genetic differences between these traits supporting the potential of a non-linear relationship between walking ability and growth rates in turkeys. Thus, using a linear regression on body or growth rate in the analysis of WA could lead to erroneous outcomes. Furthermore, assuming constant genetic correlations between growth traits and WA in multivariate analyses might also not be adequate. The severity of the impact depends on the magnitude of the variation in the genetic correlations between WA scores across growth classes. The potential genetic heterogeneity and non-linear association between WA and growth could affect ranking of birds.

## 5. Conclusions

The results of this study show that walking abilities across different growth rate classes are genetically the same trait. The findings support the hypothesis of a non-linear relationship between growth rate and walking ability, as evidenced by the significant differences in the incidence of the walking ability scores within a specific growth rate quartile and age period. Furthermore, the magnitude of these differences varies across growth quartiles and age periods. Although high, the genetic relationships between walking scores across growth rate quartiles are less than perfect. This remaining variability, if exploited in a balanced manner, is sufficient to further select for growth rate without additional deterioration in walking ability. Although a multivariate approach was used in this study, other alternatives based on smooth functions to model variation in walking ability breeding values as a function of growth rate are possible. Random regressions, reaction norms, and changepoint (breakpoint) models are appropriate approaches to manage the potential non-linear relationship between growth and mobility in turkeys.

## Figures and Tables

**Table 1:** Summary of growth rate (in kg) distribution of turkeys within three age periods.

Quartile	N	Mean	SD	Min	Max
Period 1 (0 to 12 weeks)					
1	18,690	0.080	0.003	0.036	0.085
2	18,820	0.087	0.001	0.085	0.090
3	18,802	0.092	0.001	0.090	0.095
4	18,782	0.099	0.003	0.095	0.134
Period 2 (12 to 20 weeks)					
1	18,714	0.131	0.011	0.034	0.145
2	18,662	0.152	0.004	0.145	0.158
3	18,886	0.164	0.003	0.158	0.170
4	18,832	0.181	0.009	0.170	0.550
Period 3 (0 to 20 weeks)					
1	18,734	0.102	0.004	0.075	0.107
2	18,807	0.110	0.0017	0.107	0.113
3	18,770	0.116	0.0016	0.113	0.119
4	18,783	0.124	0.004	0.119	0.228

N = Number of birds.

**Table 2:** Incidence of walking ability scores (%) across the different quartiles of the growth rate distribution for three age periods.

	1st Quartile	2nd Quartile	3rd Quartile	4th Quartile
Period 1 (0 to 12 weeks)				
CLASS 1	32.4	35.2	37.5	42.6
CLASS 2	41.2	41.7	41.8	41.0
CLASS 3	23.3	20.5	18.6	14.9
CLASS 4	2.9	2.5	1.9	1.3
Period 2 (12 to 20 weeks)				
CLASS 1	34.9	35.4	36.2	41.1
CLASS 2	39.1	41.3	43.0	42.3
CLASS 3	22.7	20.8	18.7	15.2
CLASS 4	3.2	2.3	1.9	1.1
Period 3 (0 to 20 weeks)				
CLASS 1	32.6	34.2	37.3	43.5
CLASS 2	39.3	42.0	42.6	41.8
CLASS 3	24.3	21.3	18.3	13.4
CLASS 4	3.6	2.3	1.5	1.0

CLASS 1 = birds with walking score of 1; CLASS 2 = birds with walking score of 2; CLASS 3 = birds with walking score of 3; and CLASS 4 = birds with walking scores of 4, 5, and 6.

**Table 3:** Posterior means of the residual variances for walking ability across the different growth quartiles for three growth periods.

Growth Quartile				
Period	1	2	3	4
1	0.537	0.502	0.486	0.439
2	0.558	0.503	0.483	0.439
3	0.578	0.508	0.475	0.419

**Table 4:** Heritabilities (diagonal; bold) and genetic correlations between walking abilities of birds within each of the quartiles of growth rate distribution during three age periods ^1^.

	Period 1				Period 2				Period 3			
Trait ^2^	1	2	3	4	1	2	3	4	1	2	3	4
1	**0.18**	0.89	0.87	0.84	**0.19**	0.91	0.90	0.88	**0.22**	0.90	0.88	0.86
2		**0.20**	0.94	0.86		**0.22**	0.93	0.91		**0.22**	0.91	0.89
3			**0.19**	0.94			**0.19**	0.94			**0.26**	0.94
4				**0.22**				**0.20**				**0.26**

^1^ Period 1 = 0–12 weeks; Period 2 = 12–20 weeks; Period 3 = 0–20 weeks. ^2^ walking abilities of birds in each quartile of the growth distribution within an age period were considered as different categorical traits.

## Data Availability

The data were provided by Hendrix Inc., and it is not publicly available.
